# Optimized Stem Cell Detection Using the DyeCycle-Triggered Side Population Phenotype

**DOI:** 10.1155/2016/1652389

**Published:** 2015-12-20

**Authors:** Maximilian Boesch, Dominik Wolf, Sieghart Sopper

**Affiliations:** ^1^Institute of Immunobiology, Kantonsspital St. Gallen, Rorschacherstrasse 95, 9007 St. Gallen, Switzerland; ^2^Internal Medicine V, Medical University of Innsbruck, Anichstrasse 35, 6020 Innsbruck, Austria; ^3^Medical Clinic III for Oncology, Hematology and Rheumatology, University Clinic Bonn (UKB), Sigmund-Freud-Strasse 25, 53127 Bonn, Germany; ^4^Tyrolean Cancer Research Institute, Innrain 66, 6020 Innsbruck, Austria

## Abstract

Tissue and cancer stem cells are highly attractive target populations for regenerative medicine and novel potentially curative anticancer therapeutics. In order to get a better understanding of stem cell biology and function, it is essential to reproducibly identify these stem cells from biological samples for subsequent characterization or isolation. ABC drug transporter expression is a hallmark of stem cells. This is utilized to identify (cancer) stem cells by exploiting their dye extrusion properties, which is referred to as the “*side population assay*.” Initially described for high-end flow cytometers equipped with ultraviolet lasers, this technique is now also amenable for a broader scientific community, owing to the increasing availability of violet laser-furnished cytometers and the advent of DyeCycle Violet (DCV). Here, we describe important technical aspects of the DCV-based *side population assay* and discuss potential pitfalls and caveats helping scientists to establish a valid and reproducible DCV-based *side population assay*. In addition, we investigate the suitability of blue laser-excitable DyeCycle dyes for side population detection. This knowledge will help to improve and standardize detection and isolation of stem cells based on their expression of ABC drug transporters.

## 1. Introduction

Tissue stem cells (TSC) and cancer stem cells (CSC) are of fundamental importance because they are required for tissue homeostasis/repair [1–3] and long-term tumor propagation [4–6], respectively. Thus, these cells have emerged as highly attractive target populations for regenerative medicine (TSC) and anticancer therapy (CSC), highlighting the significance of experimental procedures allowing their recovery.

It is a hallmark of both TSC and CSC that members of the ATP-binding cassette (ABC) drug transporter family are expressed. In the physiological setting, these efflux pumps serve an important purpose, as they mediate protection of TSC from harmful compounds such as xenotoxins [7]. Conversely, in the cancer setting, the cell-protective properties of ABC drug transporters facilitate the evasion of CSC from cytotoxic/cytostatic anticancer therapy, thus allowing CSC persistence despite cancer treatment potentially leading to subsequent disease recurrence [8].

ABC drug transporter expression can be exploited to functionally detect and isolate stem cells. This assay, known as* side population* (SP) analysis, makes use of dye extrusion* via* ABC drug transporters, resulting in differential fluorescence between stem and nonstem cells, which can therefore be discriminated by flow cytometry [9]. Allowing live cell recovery, SP sorting is considered a valuable tool in stem cell research and has been successfully used to purify stem cells from diverse samples such as bone marrow, tumor tissue, and cancer cell lines [10–15].

Traditionally, SP analysis has been performed using the DNA-binding dye Hoechst 33342 [10]. Although this fluorophore works well and achieves excellent resolution, it also requires an ultraviolet (UV) excitation source not commonly provided on standard flow cytometers. Vybrant DyeCycle Violet (DCV) is another DNA-binding fluorophore suitable for SP detection that in contrast to Hoechst 33342 supports violet laser excitation, thus enabling SP analysis of standard flow cytometry instruments lacking a UV laser source [16]. Importantly, the pump specificities of DCV and Hoechst 33342 are largely overlapping, indicating that the same cell populations should be detected [16, 17].

However, even though the DCV-based SP assay is increasingly used in stem cell research [14, 18], the experimental parameters influencing the discrimination of DCV-SP cells have only been insufficiently elaborated. Similarly, it is still a matter of debate which controls are most appropriate. Thus, DCV-based SP detection is often performed under suboptimal conditions and/or without adequate controls, each of which precludes the tapping of the method's full potential.

In this methods' paper, we systematically describe important experimental aspects of DCV-based SP detection. We propose that defined staining conditions as well as appropriate control selection is indispensable for the achievement of optimal results. In addition, we depict common pitfalls and how to deal with them. Our paper should help scientists to optimize DCV-based SP detection for improved performance, which is particularly relevant for the tiny stem cell populations present in tissues that inherently show a poor separation. Moreover, we also view this paper as gateway for the standardization of this useful method for stem cell sorting. Finally, we report on the suitability of recently developed blue laser-excitable cell-permeant DNA dyes to discriminate ABC transporter-expressing cells.

## 2. Results

### 2.1. Principle and Workflow of SP Detection

**Figure 1:**
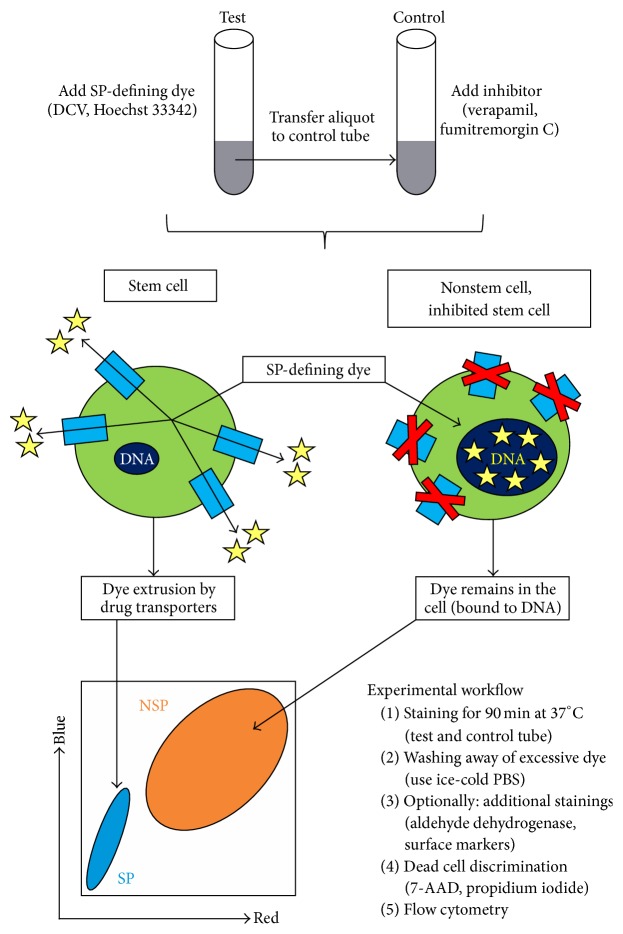
Principle and workflow of SP detection. SP-defining dyes are lipophilic and enter cells passively to target nuclear and mitochondrial DNA. However, binding to DNA occurs only in drug transporter-deficient (nonstem) cells, whereas stem cells prevent this event through transporter-mediated dye extrusion, a process that is suppressed upon compound inhibition. The resulting differential dye accumulation enables flow cytometric discrimination of stem cell-enriched SP and a corresponding NSP containing the bulk of differentiated cells. The experimental workflow and the underlying principle are depicted. Dye molecules are shown as yellow asterisks and membrane-spanning ABC drug transporters are shown as light blue rectangles. Dark blue ellipses within the cells refer to DNA in both the nuclear and mitochondrial compartment.

In SP detection, cells of interest are loaded with a lipophilic DNA-binding fluorophore (e.g., DCV, Hoechst 33342). Due to the membrane-permeant nature of such dyes, they randomly enter all cells in the sample through passive diffusion and subsequently target nuclear and mitochondrial DNA for binding (Figure 1). The differential accumulation of such dyes between stem and nonstem cells constitutes the basis of SP detection: nonstem cells lacking ABC drug transporters retain high amounts of these dyes, whereas stem cells efficiently prevent DNA-binding of the fluorophores by effluxing them right after entrance through mechanisms involving functional drug transporter activity [9]. In addition, DCV and Hoechst 33342 show a concentration-dependent change in their emission spectra (“bathochromic shift”) that mechanistically results from electronic interactions between dye molecules bound to DNA. Accordingly, when the sample is analyzed by flow cytometry using bivariate dot plots for red and blue emission, nonstem cells will have high fluorescence in both channels (due to the spectral shift particularly in the red channel), whereas the minority population of stem cells will show dim fluorescence only, appearing as the so-called SP in the lower left part of the plot (Figure 1). To finally confirm such low fluorescent cells as* bona fide* SP cells, a functional inhibitor of ABC drug transporter activity (e.g., verapamil, fumitremorgin C) needs to be included in the analysis, and SP cells must disappear upon this pharmacological intervention [9].

**Figure 2:**
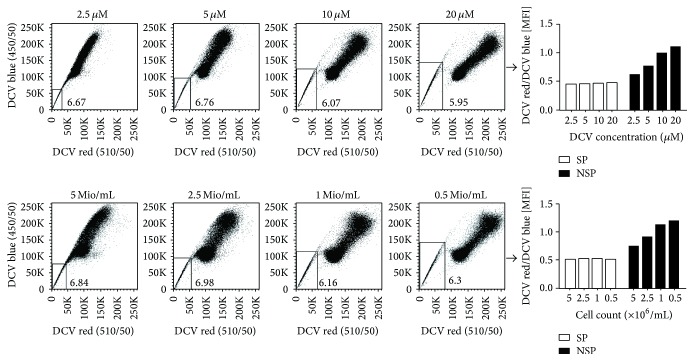
Impact of dye concentration and staining cell density on the separation of DCV-SP cells. A2780V cells were stained either with different DCV concentrations at a fixed cell density (i.e., 10^6^ cells/mL; upper panels) or with a fixed DCV concentration (i.e., 10 *μ*M) at different cell densities (lower panels). Both a high DCV concentration and a low staining cell density promote the separation of SP cells, as outlined by the increasing sizes of the rectangular gates. Note that panels “10 *μ*M” and “1 Mio/mL” correspond to each other (10 *μ*M DCV and 10^6^ cells/mL, resp.). Importantly, improved separation of SP cells at higher dye concentration or lower cell number is primarily due to increased fluorescence of NSP cells in the long-wave range of DCV emission (“DCV red”) (bar graphs).

It is important that the cells are adjusted to an defined cell count per mL and stained with an optimal amount of SP-defining dye (see Figure 2). Because drug transporter-dependent dye extrusion requires ATP and other metabolic activities, staining needs to be conducted in culture medium and at 37°C. For control purpose, aliquots of the dye-containing cell suspensions are put aside and stained in parallel in the presence of pharmacological drug transporter inhibitors (Figure 1). Importantly, the cell suspensions must be regularly agitated to ensure equal dye accessibility for all cells. After completion of staining, excessive extracellular dye is washed away by ice-cold PBS, and cells should now be chilled permanently and analyzed by flow cytometry within one hour. Although stained cells are principally stable and separation remains possible for several hours (Supplemental Figure  1(A) in Supplementary Material available online at http://dx.doi.org/10.1155/2016/1652389), immediate analysis ensures optimal cell integrity and separation and also helps to prevent gradual dye efflux from SP cells of the control tubes where active doses of the inhibitor are now lacking (Supplemental Figure  1(B)). Optionally, samples can be additionally stained for aldehyde dehydrogenase activity, another functional characteristic of stem cells, and/or cell surface markers [19, 20]. The use of a dead cell discrimination marker is strongly encouraged.

### 2.2. Staining Parameters Influencing the Separation of SP Cells

In contrast to antibodies, which are added in excess and whose required dose is primarily determined by the law of mass action, DNA-binding dyes are added at a nonexcessive amount and are very sensitive to even minor changes of dye concentration. To systematically define the dose-dependency of DCV staining, we performed SP detection of exactly the same cells using increasing dye concentrations. As illustrated in Figure 2 we found that this experimental parameter indeed has great impact on the staining outcome. While a concentration of 10 *μ*M already gives a good result, doubling of this dose to 20 *μ*M significantly improves the separation of the SP from the NSP. Conversely, when decreasing the dye concentration to 50% (5 *μ*M) or 25% (2.5 *μ*M), the staining outcome worsens significantly, with the SP becoming poorly separated especially in the long-wave range of DCV emission (“DCV red”).

In a similar experimental setup, we investigated at a given DCV concentration (i.e., 10 *μ*M) the influence of the cell count on the staining outcome. As shown in Figure 2, 10^6^ cells/mL allow a good separation of SP from NSP cells which can be further improved by decreasing the cell count (5 × 10^5^ cells/mL). Conversely, the separation becomes worse with increasing cell densities (2.5 × 10^6^ and 5 × 10^6^ cells/mL) (Figure 2) and is almost completely lost at 10^7^ cells/mL (*data not shown*). Similarly, as for the dye concentration, the cell count primarily impacts the long-wave range of DCV emission.

Another factor influencing the separation of SP cells is the incubation time, that is, the duration of dye exposure. For most applications, a staining duration of 90 min is adequate, as deducible from numerous publications [10, 13–15]. Corroborating this notion, it has been shown for Hoechst 33342 that shortening or extending the staining duration can lead to a high variation regarding the size of the detected SP [21, 22]. Here, we have revisited this issue and looked at SP/NSP separation after different staining times (i.e., 30, 60, 90, and 120 min). We found that, at least in the cell line model used here (A2780), a staining duration of 30 min does not allow for optimal dye accumulation in NSP cells, even though dye extrusion by SP cells is already “saturated” at this point (Supplemental Figure  2). Conversely, we could not detect any notable differences in the staining patterns after 60, 90, and 120 min, except that the NSP peak might get a little sharper with prolonged dye exposure (Supplemental Figure  2). However, because extending the incubation time can significantly affect cell viability which is particularly relevant for primary cell samples and control aliquots where a pharmacological inhibitor is additionally impinging on the cells, we recommend adhering to the “default” incubation time of 90 min and adapt it only upon demand in particular cases.

Together, our experiments show that, unlike antibodies which are less susceptible to changes in concentration or cell density, SP-defining dyes such as DCV are extremely sensitive to both parameters. Based on these results, we suggest that staining of 10^6^ cells/mL with 10 *μ*M of DCV is a good compromise between performance, dye consumption, and recordable cell count in analysis experiments. For sorting applications with the goal of high cell yield, we suggest to increase the cell count depending on the size of the respective SP (a larger SP gives a higher cell yield and therefore does not require as many input cells), but we would refrain from exceeding 5 × 10^6^ cells/mL due to increasingly poor separation. Anyway, it is recommended to determine the best suitable cell dose and dye concentration for each particular cell type/application separately. Regarding the time of dye exposure, researchers can be confident that 90 min is optimal for most cell types/samples.

### 2.3. Specific Gating Considerations in SP Analysis

Excluding unwanted cells (or “events”) from the final analysis is essential for obtaining optimal results in flow cytometry experiments. For many applications including SP detection, the proposed gating strategy consists of exclusion of debris, doublets, and dead cells, based on forward/side scatter characteristics and staining with a dead cell discrimination marker. Although this procedure seems obvious, we would like to stress two SP-specific peculiarities.

**Figure 3:**
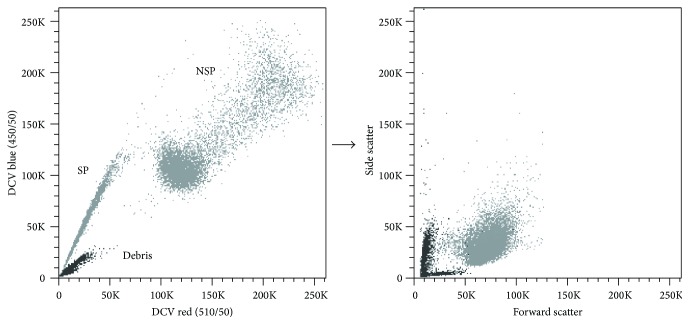
Characteristic localization of debris close to the SP. A2780V cells were stained with 10 *μ*M DCV (10^6^ cells/mL) and analysed by flow cytometry. In this particular case, debris was not properly excluded in the initial forward/side scatter gate to illustrate its location relative to SP and NSP. Debris generally tends to generate a “smear population” in the lower left part of the plot that often shows overlap with the* bona fide* SP. Thus, debris exclusion by adequate forward/side scatter gating is really crucial in SP analysis.

Namely, we found a strong tendency of debris (possibly also of 7-AAD-negative early apoptotic cells and apoptotic bodies with insufficient DNA content to qualify as 7-AAD-positive) to cluster adjacent to the SP which can hinder the definition of SP cells (Figure 3). Although the authenticity of SP is controllable using pharmacological inhibition, debris can still be interfering, particularly when the populations are overlapping as it is frequently the case when analyzing primary samples. To counteract this potential pitfall, we suggest a “stricter-than-normal” forward/side scatter gating strategy and also recommend increasing the forward scatter threshold rate.

Furthermore, even though for optimal results doublets should definitely be excluded (e.g., by comparing the height and width signals of forward scatter), we would like to highlight that from a technical viewpoint doublets should not be interfering with the correct identification of SP cells. This is because SP-SP doublet will still show up in the SP gate whereas NSP-NSP doublet cannot apparently become “SP event.” Similarly, SP-NSP doublet will under normal conditions locate to the NSP region. Thus, although certainly not recommended, no (or improper) exclusion of doublets should not lead to significant cross-contamination of the SP by NSP cells.

### 2.4. SP Inhibition Using Pharmacological Compounds

**Figure 4:**
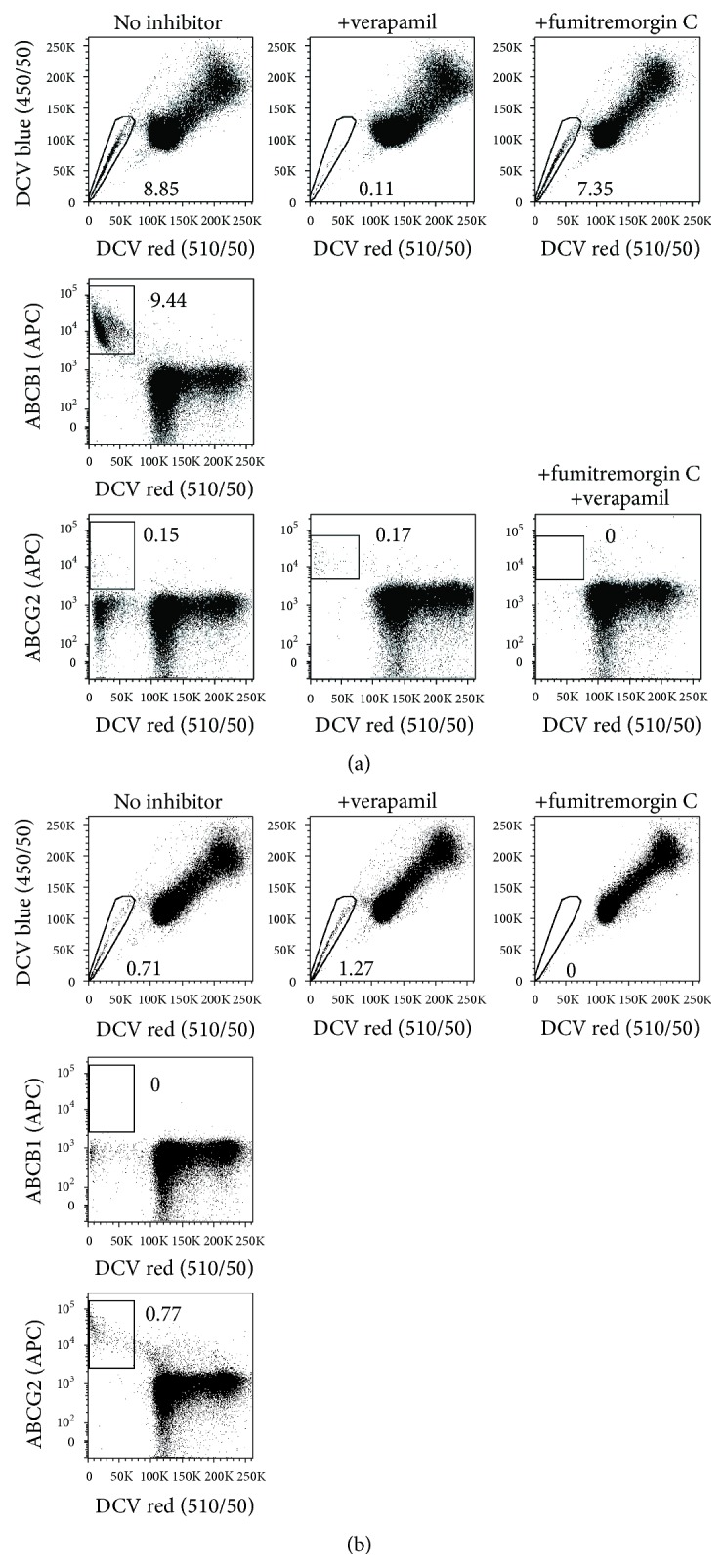
Characterization of DCV-SP cells using pharmacological inhibition and antibody-based costaining of SP-conferring drug transporters. A2780V cells containing ABCB1-positive SP (a) and parental A2780 cells harbouring an ABCG2-positive SP (b) were stained with 10 *μ*M DCV (10^6^ cells/mL) and subjected to a set of control experiments. Specifically, the samples were inhibited using 50 *μ*M verapamil (blocking several drug transporters) or 20 *μ*M fumitremorgin C (blocking ABCG2 specifically) or, where indicated, both. In addition, noninhibited and inhibited cells were costained for the drug transporters ABCB1 and ABCG2 using respective monoclonal antibodies. This multimodal approach helped us to better define particularly A2780V cells, whose SP contains a major population of ABCB1-positive cells (~8–10%) and a minor population of ABCG2-expressing cells (~0.1%). Note that, at least in our model systems, verapamil does not inhibit the activity of ABCG2.

**Figure 5:**
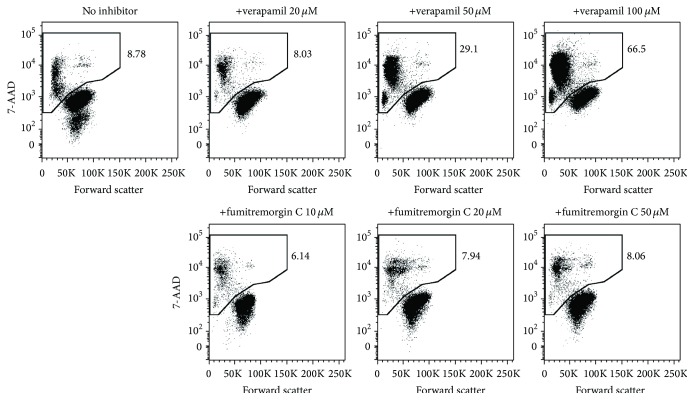
Evaluation of cell death induction by pharmacological inhibitors of drug transporter activity. A2780V cells (10^6^ cells/mL) were stained with 10 *μ*M DCV. Control aliquots containing different concentrations of inhibitor (either verapamil or fumitremorgin C) were prepared thereof and analysed for cell viability using 7-AAD staining. Verapamil induces significant cell death already at the commonly used dose of 50 *μ*M, whereas the ABCG2-specific inhibitor fumitremorgin C leaves the cells virtually unaffected.

Sensitivity to pharmacological drug transporter inhibitors is a key criterion of* bona fide* SP cells [9, 19]. Although various inhibitors are available (several of which have been specifically designed for adjuvant treatment of drug-resistant cancers), verapamil, fumitremorgin C, and reserpine are among the most commonly used compounds for SP blockage. Verapamil and reserpine, which are clinically approved therapeutics, block several ABC drug transporters [19], whereas fumitremorgin C is considered a specific inhibitor of ABCG2 [9, 23]. Although this inhibition profile would primarily argue for the use of verapamil and reserpine particularly in initial screenings, these substances also have major drawbacks. For instance, reserpine, but not verapamil and fumitremorgin C, shows significant autofluorescence in the wavelength range of DCV emission (Supplemental Figure  3), which potentially leads to false-positive detection of SP. Thus, we would refrain from using reserpine as inhibition control for DCV-defined SP. Further, our data suggest that verapamil, which potently blocks ABCB1, ABCB5, and ABCC1, does not or hardly inhibit ABCG2 at commonly used concentrations (Figure 4), therefore “sparing” ABCG2-positive SP cells. On these grounds, the sole use of verapamil does not seem to be a good option particularly when the SP-conferring drug transporter is unknown. Another, more general, point that needs to be considered is that drug transporter inhibitors, especially verapamil, can quickly induce cell death (Figure 5) which might interfere with the inhibition and, hence, definition of SP cells. Thus, a dead cell discrimination marker such as 7-AAD or propidium iodide should definitely be used.

Altogether, we suggest a dual inhibition strategy using both verapamil and fumitremorgin C. Although these inhibitors can be combined in a single tube (Figure 4(a)) [9], the use of separate tubes holds the advantage of deciphering whether ABCG2 is involved in conferring the SP phenotype, based on the selectivity of fumitremorgin C towards this transporter. Importantly, this dual inhibition strategy seems to be reasonable also in view of the fact that most of the published SP express ABCG2 and/or ABCB1 [11, 13, 24–28]. Regarding the optimal working concentrations of verapamil and fumitremorgin C, it is our experience that the commonly used doses (20–50 *μ*M for verapamil and 10–20 *μ*M for fumitremorgin C) work well using the cell concentrations described above and should not be escalated to avoid additional cell death induction (Figure 5).

### 2.5. Combinatorial Staining Approaches

Sometimes, it can be difficult to demonstrate without doubt the presence of SP cells in a sample. Two of the most common reasons for this are (i) poor separation from the NSP due to low drug transporter activity in the SP and (ii) insensitivity of the SP-conferring drug transporter to the used inhibitor(s). Moreover, certain cell types are inherently hypersensitive to drug transporter inhibitors and, therefore, might not be amenable to appropriate inhibition experiments at all [29]. In such cases, surface staining of ABC drug transporters by respective antibodies can provide a valuable tool since a second specificity is added to the actual SP analysis. Figure 4(a) illustrates how ABC drug transporter staining helped us to better define the SP of the A2780V ovarian cancer cell line: the inhibitor profile—sensitivity to verapamil but insensitivity to fumitremorgin C—argued for expression of a drug transporter other than ABCG2, which we then confirmed using staining with anti-ABCB1 antibody. However, even though the bulk of SP was responsive to verapamil, a small population of cells (~0.1%) remained unaffected and stayed in the SP gate. Using staining with an anti-ABCG2 antibody, we finally disclosed these cells as ABCG2-expressers. Thus, staining with ABC drug transporters helped us to uncover the presence of two distinct SP (i.e., a predominating ABCB1-positive and a minor ABCG2-positive population) within a single cell line. In contrast, when SP can be completely blocked with the ABCG2-specific inhibitor fumitremorgin C, staining for ABC drug transporters does not provide additional information but can be used for double-checking-purpose (Figure 4(b)). Collectively, costaining for ABC drug transporters not only may improve the resolution and/or increase the information content of the analysis but also can serve as important control particularly when inhibition experiments do not give a clear result. Furthermore, identifying the SP-conferring drug transporter can provide useful cues on the functional properties of the investigated cells. For instance, ABCG2, ABCB1, and ABCB5, which all confer the SP phenotype and are linked to tumor stemness [7, 11–13, 27, 30], have different substrate specificities and therefore mediate a different resistance profile [8, 31, 32]. This can have different clinical implications depending on the respective tumor type and the associated therapeutic modality.

It is also important to note that DCV-based SP detection not only is compatible with green and red laser-excitable fluorochromes but also can be performed in conjunction with blue laser-excitable dyes emitting in the FITC (530 nm) and PE (585 nm) channels, adequate compensation provided. This is particularly relevant for applications in which SP cells are to be additionally tested for activity of the stem cell marker aldehyde dehydrogenase (detected at 530 nm) or when SP analysis shall be performed with GFP/YFP-tagged samples. Moreover, the compatibility of DCV with most other fluorochromes and channels is crucial for proper marker-based discrimination of the target population in complex cell samples such as dissociated tissue (markers often used for this purpose include EpCAM, CD45, CD31, and Ter-119). As an example, it was recently shown that the SP of freshly isolated human glioblastoma samples was established by CD31-positive brain endothelial cells, rather than tumor cells, resulting in nontumorigenicity of the detected SP [33]. Hence, to draw the right conclusions on the functional characteristics of identified SP, costaining with discriminating antibodies is absolutely mandatory, particularly in the case of highly heterogeneous samples.

Combinatorial stainings involving SP detection require a particular succession (Figure 1). Staining for SP comes first, followed by aldehyde dehydrogenase staining (optionally), in turn followed by antibody-mediated surface/CD marker staining (optionally). The last step prior to flow cytometry should always be dead cell discrimination (e.g., using 7-AAD or propidium iodide). The detailed experimental protocol for such combined measurements can be looked up in the excellent review articles of Greve and coworkers [19] and Telford [20] and is not separately depicted here.

### 2.6. DCV Does Not Compromise Cell Viability and Function

**Figure 6:**
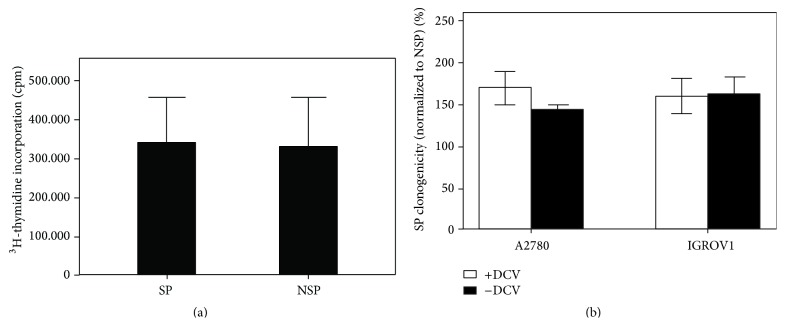
DCV does not impinge on cell proliferation or single cell clonogenicity. (a) A2780V cells were stained with 10 *μ*M DCV (10^6^ cells/mL) and SP/NSP fractions were flow sorted. Equal numbers of cells were then cultured and cell proliferation was assessed after 48 h using ^3^H-thymidine incorporation. Under these conditions, dye-retaining NSP cells proliferate equally well as their dye-extruded SP counterparts. (b) Comparative single cell cloning of freshly sorted (“+DCV”)* versus* presorted,* in vitro* expanded SP/NSP cells (“−DCV”). In two investigated cell lines (i.e., A2780 and IGROV1), no notable differences could be detected between the two experimental setups, indicating that the presence of DCV does not affect the clonogenic capacity of NSP cells.

A major concern among stem cell biologists has been potential toxicity of SP-defining dyes [9]. Although this point is irrelevant for sole analysis experiments, it is of fundamental importance for functional investigation of sorted cells, especially when stem cell properties such as clonogenicity and engraftment are tested. This is because, owing to the differential dye accumulation, toxicity would primarily affect the NSP, thereby generating artefacts in favor of the SP. To test whether DCV is toxic to cells (Hoechst 33342 and other SP-defining dyes are beyond the scope of this study), we freshly sorted DCV^dim^ (SP) and DCV^bright^ (NSP) cells and analyzed their proliferation rate using ^3^H-thymidine incorporation. Using this approach, we could not detect any notable impairment in the proliferation of NSP cells (Figure 6(a)). Furthermore, we performed single cell cloning with presorted SP and NSP fractions in which the SP-defining dye (i.e., DCV) was diluted out over weeks of culture. When comparing these data with those of freshly sorted SP and NSP cells [13], no appreciable differences in the clonogenicity of either cell population (SP or NSP) were found between the two experimental setups in independent cell line models (Figure 6(b)). Based on these observations, we conclude that DCV has no relevant toxicity at least at the concentration generally used for cell sorting (i.e., 10 *μ*M). Finally, it has also been shown that Hoechst 33342 dose-dependently downregulates particular stem cell-associated genes including Sox17 and EPC1, which might differentially affect the functional properties of SP and NSP cells [34]. In a microarray analysis of DCV-defined SP and NSP cells, however, we did not find such effects, as both Sox17 (*p* = 0.85) and EPC1 (*p* = 0.99)—as well as further stem cell-related genes—showed equal expression in both cell fractions (Supplemental Figure  4). Thus, although very subtle toxicity or minimal gene-regulatory activity can never be totally excluded (nor in the case of other methods for stem cell sorting such as aldehyde dehydrogenase or antibody staining), we are confident that DCV-based SP sorting is a valid tool not only for stem cell detection and isolation but also for comparative functional investigation of stem* versus* nonstem cells.

### 2.7. Suitability of Different Flow Cytometers for SP Detection

**Figure 7:**
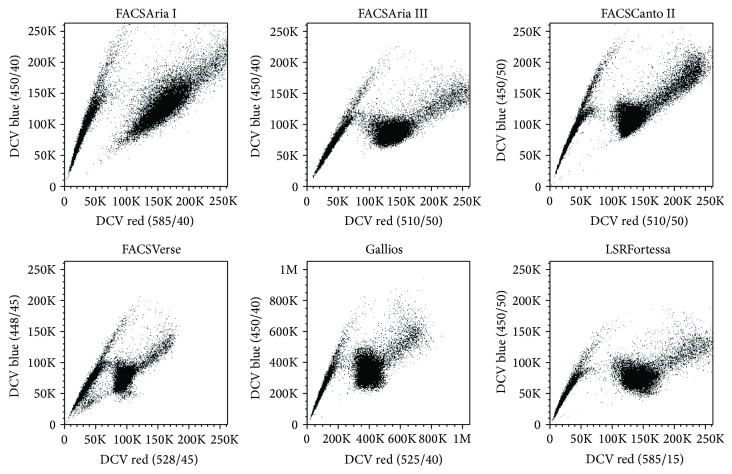
Detection of DCV-SP cells using various flow cytometric instruments. A2780V cells were stained with 10 *μ*M DCV (10^6^ cells/mL) and analysed on the indicated flow cytometers within one hour. Note that all of the investigated instruments facilitate reasonable discrimination of SP cells, even though certain instruments separate particularly well, for instance, LSRFortessa.

Different flow cytometers are equipped with different optics and fluidics systems, and they further differ in the geometry of their cuvette flow cell. Considering that DNA-binding fluorophores measured in linear mode are particularly susceptible to such parameters, it is well conceivable that certain instruments are better or worse suited for SP detection. To address this point, we consecutively analyzed the same DCV-stained samples on different flow cytometers ranging from Beckman Coulter instrument (i.e., Gallios) to a set of Becton Dickinson instruments (i.e., FACSCanto II, FACSVerse, LSRFortessa, FACSAria I, and FACSAria III). We found that the SP could be sufficiently discriminated on all instruments, even though specific flow cytometers such as LSRFortessa and FACSCanto II seem to be particularly suitable for this purpose mostly because of improved separation in the long-wave range of DCV emission (“DCV red”) (Figure 7). We also observed that among the sorting instruments the FACSAria III gives a slightly better separation than its predecessor model (i.e., FACSAria I), and this is likely because of the differently shaped cuvette flow cell. Nonetheless, despite these minor differences, we conclude that for most applications the investigated instruments are equally suited and that the prime limiting factor for the separation of SP cells is indeed the staining process itself. Of note, the red fluorescence of Hoechst 33342 [10] and DCV [16] has traditionally been measured using long-pass filters above 600 nm. However, in some of the instruments used for comparison, only band-pass filters with shorter wavelengths are installed which cannot be exchanged easily. Nevertheless, we found a good separation between SP and NSP even with the filters of Gallios, FACSCanto II, and FACSVerse instruments (Figure 7). Indeed, when comparing the separation using different filters on LSRFortessa, no appreciable differences were observed (Supplemental Figure  5). Thus, it is possible to perform SP analysis with DCV also on instruments with just two detectors for the violet laser without the necessity to change filters.

### 2.8. SP Detection Using DCG and DCO

**Figure 8:**
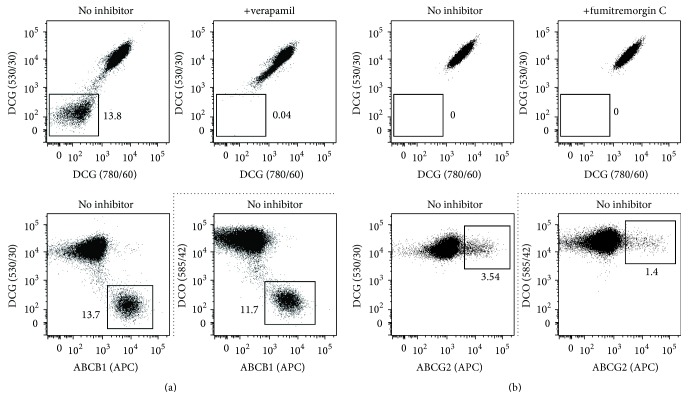
SP detection using the second-generation DyeCycle dyes DCG and DCO. A2780V cells harbouring an ABCB1-positive SP (a) and parental A2780 cells containing an ABCG2-positive SP (b) were stained with 500 nM of either DCG or DCO (10^6^ cells/mL) and analysed by flow cytometry using the indicated emission filters (DCO data accentuated by dotted lines). For control purpose, verapamil (A2780V) or fumitremorgin C inhibition (A2780) was performed, and drug transporters were stained with APC-conjugated monoclonal antibodies. Both DCG and DCO are able to detect ABCB1-positive SP cells, whereas neither dye can identify SP cells that express ABCG2. Note the extreme separation of ABCB1-SP cells, which made it impossible to measure the fluorescence linearly.

DCG and DCO are second-generation DyeCycle dyes that, in contrast to DCV, support blue laser excitation, hence allowing analysis also on flow cytometers lacking UV and violet laser sources [35]. However, since DCG and DCO are primarily used for cell cycle analysis, their susceptibility to efflux* via* drug transporters has not been elaborated so that their applicability as SP-defining dyes remains currently unknown. Thus, we performed DCG and DCO staining of cell lines known to harbor ABCG2- (A2780) and ABCB1-positive SP (A2780V). These experiments revealed that both DCG and DCO are able to detect ABCB1-positive SP (Figure 8(a)), whereas neither dye was able to detect ABCG2-positive SP (Figure 8(b)) (the presence of ABCB1- or ABCG2-expressing cells was also confirmed using costaining with respective antibodies). However, due to extreme separation of SP cells from NSP cells, we were unable to record the data in linear mode even after downtitration of the dyes and were thus forced to switch to logarithmic scaling, which might also have precluded the typical appearance as a* side population* (Figure 8(a)). Altogether we conclude that, from a technical viewpoint, SP discrimination by DCG and DCO is quite different from “classical” SP detection using DCV or Hoechst 33342. Although the great difference in DCG/DCO fluorescence between ABCB1-positive and ABCB1-negative cells indicates a better sensitivity of these recently developed dyes, their broad emission spectrum is largely incompatible with additional blue laser parameters, hence restricting costainings to red laser-excitable fluorochromes. Another fundamental limitation is certainly the fact that neither DCG nor DCO can detect the ABCG2 efflux pump, which is considered the molecular determinant of numerous, possibly of the majority of, SP compartments [7, 11, 13, 14, 28]. Thus, we reason that DCG and DCO will only play a minor role in SP analysis, despite their advantage of being compatible with virtually any flow cytometry instrument on the market.

## 3. Discussion

TSC and CSC are considered to hold immense therapeutic potential. For instance, lack of sufficient and/or appropriate tissue repair is the defining hallmark of several injury-related or degenerative medical conditions, and therapeutic supply of stem cells (e.g., neural stem cells, mesenchymal stem cells, and intestinal stem cells) is regarded as an attractive treatment option for such patients [36–38]. Conceptually, these approaches are based on the well-known ability of TSC for lifelong demand-adapted regeneration of all cell types within a particular organ or tissue. Conversely, in the cancer setting, stem cells have adverse effects, fueling tumor growth and contributing to metastatic seeding during progression [4–6, 39]. Moreover, due to intrinsic resistance to both cytotoxic and targeted anticancer drugs, CSC can also survive the primary therapeutic intervention, which predisposes the patient to relapse [8]. Thus, novel therapeutic modalities and at best CSC-selective drugs are desirable to allow eradication of these cells during primary treatment. However, to translate novel stem cell-directed treatment concepts into clinical practice, a more thorough understanding of stem cell biology and function will be necessary. To this end, we will have to find means enabling the reproducible identification and isolation of TSC and CSC from biological samples such as cell lines and, most importantly, patient specimens, and this is in the setting of different organ systems (TSC) and various tumor entities (CSC).

The SP assay provides a powerful method to functionally define TSC and CSC by ABC drug transporter-dependent dye extrusion and complements other stem cell identification methods that are based on either antibody-mediated staining of stem cell-associated surface markers (e.g., CD24, CD34, CD44, CD90/Thy-1, CD105/endoglin, CD117/c-kit, CD133/prominin-1, CD166, CD326/EpCAM, Lgr5, and Sca-1) or detection of stem cell-related enzymatic activity (i.e., aldehyde dehydrogenase) [3, 19, 40]. A major advantage of SP analysis is certainly the high conservation of ABC drug transporters in TSC and CSC, which implicates that the SP phenotype should be a robust marker in both physiological and pathological settings. In contrast, surface stem cell markers often show tissue-specific expression, which might preclude broad application. Corroborating this notion, we recently found that, of several CSC markers tested, only the SP phenotype robustly identified distinct small subsets of cells with stem cell properties throughout various ovarian cancer cell lines [13]. Another advantage of SP analysis is that,* via* active dye efflux, more than one type of drug transporter is recognized (e.g., ABCG2, ABCB1, and ABCC5), hence allowing the identification of different stem cell populations [11, 13, 27, 28, 30, 41]. Finally, it was also shown that detection of functional ABC drug transporter activity yields higher stem cell selectivity and/or resolution as compared to immunophenotyping of SP-conferring drug transporters [42]. Together, these considerations highlight the suitability of SP detection for identification and isolation of drug transporter-positive stem cells from diverse tissues and tumor types. Moreover, reasoning that disease recurrence and refractoriness to therapy are the major obstacles in clinical oncology, we believe that methods allowing the identification of cells with direct implications in drug resistance (e.g., SP cells) are of particular significance, also in view of the development of novel therapeutics meeting those clinical needs. However, in this context, care has to be taken that* bona fide* CSC are not confused with multidrug-resistant bulk tumor cells which can also express high amounts of drug transporters but which will lack defining features of stem cells such as tumor-sustaining potential [17]. Similarly, in the physiological setting, it should be kept in mind that drug transporter expression consistent with SP appearance will not be exclusive to stem cells either but may also be found in certain differentiated cell types, especially endothelial cells at crucial body sites such as the blood-brain-barrier, the intestine, and the placenta [7, 33].

The implementation of DCV as a violet laser-excitable Hoechst 33342 alternative has enabled researchers to perform SP detection also on standard flow cytometers devoid of UV laser source [16]. Meanwhile, DCV-based SP analysis has been successfully used to identify and isolate stem cells from various tissues and tumor types [13, 14, 18], yet, paradoxically, the experimental parameters influencing the detection and/or separation of DCV-SP cells have never been systematically investigated. Here, we addressed this question and performed a set of experiments to illustrate very basic properties of DCV-based SP detection on the one hand and highlight ways to optimize this functional assay on the other.

Apart from cell-intrinsic factors such as the effective activity of the expressed ABC drug transporter(s), a proper separation of DCV-SP cells primarily depends on a low cell density (≤10^6^ cells/mL) and/or a high dye concentration (≥10 *μ*M), as well as an accurate gating strategy (i.e., exclusion of debris, doublets and dead cells). Other factors influencing the staining outcome and/or the size of the detected SP include the duration of incubation [21, 22] and culture conditions such as the time after splitting, the degree of confluency, and the amount of glucose in the medium [43–45]. Once putative SP has been found, it needs to be confirmed using inhibition with pharmacological compounds and/or concomitant staining with antibodies specific for ABC drug transporters. Regarding inhibition, we suggest a dual strategy using verapamil and fumitremorgin C, so as to be sure to cover the most important SP-conferring transporters including ABCB1, ABCB5 (both verapamil sensitive), and ABCG2 (fumitremorgin C sensitive). In contrast, the use of reserpine is not recommended due to autofluorescence in the relevant wavelength range, potentially interfering with the DCV signal.

Of note, DCV is also broadly applicable. For instance, this dye is compatible with most fluorochromes including blue laser-excitable FITC and PE (and its tandem derivatives), which is particularly relevant for multicolor applications of primary tissue samples that are inherently heterogeneous and thus require the definition of principal cell populations based on surface markers (e.g., EpCAM, CD45, and CD31). Moreover, our study demonstrated that DCV can be analyzed on most flow cytometers—and sorters commonly in use including Gallios, FACSCanto II, FACSVerse, LSRFortessa, FACSAria I, and FACSAria III instruments. This finding was rather unexpected considering the partially considerable differences in fluidics systems and flow cell geometry and optics (especially the lack of filters originally used for SP detection) between instruments and the susceptibility of DNA-binding dyes to such parameters. Compatibility with blue laser excitation even further broadens the list of possible instruments for the second-generation DyeCycle dyes DCG and DCO, but here the limitation is faced that neither dye can detect ABCG2-expressing cells. Hence, the use of these dyes is inherently restricted to a subfraction of SP expressing ABCB1.

An important issue in SP analysis is the question whether SP-defining dyes, which bind to DNA, are cytotoxic or gene-regulatory to NSP cells and hence would generate artefacts in functional studies. Hoechst 33342, for example, has been reported to dose-dependently downmodulate particular genes including a fetal stem cell factor (i.e., Sox17) and a Polycomb group gene (i.e., EPC1), and it was hypothesized that these effects could imprint on the global expression profile of NSP cells [34]. Here, we have scrutinized potential toxic effects of DCV on the level of both proliferation and clonogenicity. Our results showed no detectable toxicity of the dye at least at the concentrations used for cell sorting. Furthermore, in a microarray analysis of DCV-defined SP and NSP cells, we found that neither Sox17 nor EPC1 was downregulated in the NSP, indicating that, unlike Hoechst 33342, DCV does not impinge on the expression of stemness-related genes. We are therefore confident that DCV-based SP analysis provides a sound methodology not only for the detection of stem cells, but also for their isolation and testing in functional assays.

Collectively, we believe that DCV-based SP analysis is a valuable—but still undervalued—method for stem cell identification and purification that complements antibody-based approaches and detection of stem cell-specific enzymes (i.e., aldehyde dehydrogenase). On the one hand, the power of the SP assay is to be seen in the operating mode itself, because stem cells are identified based on a* functional* parameter, rather than sole antigen expression, which can result in increased stem cell selectivity. On the other hand, it is certainly also an advantage that ABC-family efflux pumps are highly conserved among stem cell populations and that the SP assay can detect several transporters including ABCB1 and ABCG2. We reason that these facts make the SP assay appealing particularly to cancer researchers aiming at disclosing treatment-refractory subpopulations and developing novel therapeutics against them. Finally, the implementation of DCV and the thenceforth compatibility with violet laser excitation have meant a major step forward in SP analysis, which can now be performed in most laboratories worldwide. In face of this increasing global use, we hope that our study can provide helpful advice to stem cell researchers and contribute to the improvement and standardization of the DCV-based SP assay.

## 4. Materials and Methods

### 4.1. Cell Lines and Culture Conditions

The A2780 human ovarian carcinoma cell line was purchased from Sigma-Aldrich (Catalog number 93112519), and A2780V cells, a subline of A2780 [46], were kindly obtained from Professor Zeillinger, Vienna, Austria. IGROV1 ovarian carcinoma cells [47] were generously provided by Professor Brown, London, UK. All cell lines were grown in RPMI 1640 medium (Gibco, Catalog number 11875-093) supplemented with 10% FBS (Biochrom, Catalog number S0615), 2 mM L-glutamine (Catalog number 25030-081), and 1x penicillin/streptomycin (Catalog number 15070-063) (both from Gibco). Cells were cultured under standard conditions (i.e., 37°C and 5% CO_2_) and split before exceeding 90% confluency. A2780 cells have ABCG2/Bcrp1 (CD338) positive SP of roughly 1%, whereas A2780V and IGROV1 cells have ABCB1/P-glycoprotein (CD243) positive SP of roughly 10% and 1%, respectively [13].

### 4.2. SP Detection

SP detection was performed as described previously [13, 17]. Briefly, cells were harvested and adjusted in fresh medium to a certain cell count per mL (between 5 × 10^5^ cells/mL and 1 × 10^7^ cells/mL). A certain amount of DCV (Molecular Probes, Catalog number V35003) was added (between 2.5 *μ*M and 20 *μ*M) and the suspension was vortexed to ensure equal distribution of the dye. Aliquots thereof were put aside for control purpose, and either verapamil (20–100 *μ*M) (Catalog number V4629), fumitremorgin C (10–50 *μ*M) (Catalog number F9054), or reserpine (20–100 *μ*M) (Catalog number 83580) was added (all inhibitors were from Sigma-Aldrich). Verapamil and reserpine are known to block several ABC drug transporters [19], while fumitremorgin C specifically inhibits drug transport* via* ABCG2 [9, 23]. Unless otherwise specified, test and control tubes were incubated for 90 min in the dark at 37°C, with gentle agitation every 15 min. After completion of staining, extracellular DCV was washed away in >10x volume ice-cold PBS (Gibco, Catalog number 10010-023), and the cell pellet was resuspended in 200 *μ*L ice-cold PBS. To discriminate dead cells, 7-AAD (BD Pharmingen, Catalog number 559925) was added according to the manufacturer's instructions. Samples were chilled on ice and immediately analyzed by flow cytometry.

In the case of SP detection using blue laser-excitable second-generation DyeCycle dyes, 1 × 10^6^ cells/mL were stained with Vybrant DyeCycle Green (DCG) (Catalog number V35004) or Vybrant DyeCycle Orange (DCO) (Catalog number V35005) (both from Molecular Probes). DCG and DCO were each added at the pretitrated concentration of 500 nM, and staining was performed exactly as described above. In the case of DCO, dead cells were discriminated by staining with DAPI (Molecular Probes, Catalog number D1306).

### 4.3. Antibody Costaining

On some occasions, DCV/ABC drug transporter costainings were performed [13, 19]. To this end, DCV-prestained cells were washed in >10x volume ice-cold PBS and stained for 30 min at 4°C with APC-conjugated monoclonal antibodies directed against ABCG2 (clone 5D3) (BioLegend, Catalog number 332020) or ABCB1 (clone UIC2) (eBioscience, Catalog number 17-2439-42). Thereafter, excessive antibody was washed away in >10x volume ice-cold PBS, and the cells were stained with 7-AAD as described above. Antibodies were added at optimized concentrations and staining specificity was controlled using an irrelevant antibody (anti-CD3, clone UCHT1) (BioLegend, Catalog number 300412).

### 4.4. Flow Cytometry

Flow cytometric analyses and cell sorting were performed on FACSCanto II and FACSAria I instrument, respectively (both from BD Biosciences). On two occasions, cells were additionally analyzed on FACSVerse, LSRFortessa, FACSAria III (all from BD Biosciences), and Gallios instrument (Beckman Coulter). DCV fluorescence was measured in linear mode, whereas the other fluorescence (i.e., DCG, DCO, 7-AAD, DAPI, and antibodies) was measured logarithmically. A minimum of 100.000 events was recorded.

Data were analyzed using FlowJo version 9.6 (TreeStar). Debris was excluded based on forward/side scatter characteristics and doublets were discriminated using the height and width signals of forward scatter. Living (7-AAD- or DAPI-negative) cells were finally analyzed on bivariate dot plots for the indicated parameters.

### 4.5. Cell Proliferation

Proliferation of DCV^dim^ (SP) and DCV^bright^ (nonside population, NSP) cells was measured using ^3^H-thymidine incorporation. To this end, freshly sorted cells were seeded in 96-well plates at a density of 2 × 10^4^ cells/well/150 *μ*L culture medium and allowed to grow for 48 h. Cells were then pulsed for 16–18 hours with 2 *μ*Ci ^3^H-thymidine (Hartmann Analytic, Catalog number MT 846). The degree of ^3^H-thymidine incorporation was determined using scintillation counting of beta rays.

### 4.6. Clonogenic Assay

To investigate whether DCV impacts the clonogenic potential of cells, freshly discriminated (10 *μ*M DCV; “+DCV”) [13] or established (i.e., presorted,* in vitro* expanded; “−DCV”) SP and NSP fractions were single cell-sorted into 96-well plates containing 150 *μ*L medium and incubated for two weeks. The number of wells with growing clones was quantified under a bright-field microscope and compared between the two experimental setups. Data are shown as percentage SP clonogenicity, with the clonogenicity of the corresponding NSP set to 100%.

### 4.7. Statistical Analysis

Unless otherwise stated, flow cytometry data are representative examples of three or more independent experiments. Quantitative results are shown as mean ± SEM of at least three independent single values. Statistical significance of quantitative results was determined using a two-sided Student's *t*-test for paired samples. *P* values < 0.05 were considered significant.

## Supplementary Material

Supplemental Figure 1: Stability of DCV-Stained Samples. (A) SP-enriched A2780 cells were stained with 10 µM DCV (106 cells/ml) and kept on ice in the dark for 0, 6 and 22 h prior to flow cytometry. Even though cell death is increased after 6 and 22 h (data not shown), the staining itself (i.e., the separation of SP from NSP cells) is stable over the investigated time period. (B) Kinetic analysis of the same cells inhibited with 20 µM fumitremorgin C. Note that after washing, SP cells gradually re-appear owing to the henceforth lack of active inhibitor.
Supplemental Figure 2: SP/NSP Separation as a Function of Staining Duration. SP-enriched A2780 cells were stained with 10 µM DCV (106 cells/ml) for the indicated periods of time and analysed by flow cytometry. A staining duration of 30 min does not permit optimal dye accumulation in NSP cells, as detectable particularly in the long-wave range of DCV emission (‘DCV red'). Conversely, there is not much difference in staining outcomes between 60, 90 and 120 min, except that the NSP peak is somewhat sharpened with longer dye exposure. Altogether, and considering the heterogeneity in dye accumulation kinetics and cell death induction between different cell types, we propose a ‘default' staining duration of 90 min, which can of course be adapted upon demand.
Supplemental Figure 3: Autofluorescence of Reserpine in the DCV-Relevant Wavelength Range. In the absence of DCV, A2780V cells were incubated for 90 min at 37°C with either no inhibitor, 50 µM verapamil, 20 µM fumitremorgin C, or 50 µM reserpine. Thereafter, cells were washed and analysed by flow cytometry for emission in the ‘DCV blue' (450/50) and ‘DCV red' (510/50) channels (note that due to the lack of DCV in the analysis, the detector voltages had to be increased accordingly). In contrast to verapamil and fumitremorgin C which are non-excitable by the violet laser, reserpine shows significant autofluorescence which might potentially interfere with the DCV signal, therefore making this compound less suited for DCV-based SP detection.
Supplemental Figure 4: Sox17 and EPC1 Expression in DCV-Defined SP/NSP Fractions. A2780V cells were stained with 10 µM DCV (2.5x106 cells/ml) and SP and NSP fractions were flow sorted (n=3). RNA was isolated and samples were processed for microarray analysis performed on the GeneChip® Human Gene 1.0 ST Array platform (Affymetrix). Data were normalized and bio-informatically analysed for differential gene expression as defined by M ≥±1 (representing a fold-change of ≥2) and p ≤0.05. Sox17 and EPC1, two stem cell-related genes downmodulated by Hoechst 33342, did not show underrepresentation in NSP cells, indicating that these genes are not regulated by DCV.
Supplemental Figure 5: Detection of DCV-SP Cells Using Different Filter Combinations. A2780V cells (106 cells/ml) were stained with 10 µM DCV and analysed on an LSRFortessa using the indicated filters. Evidently, all of the investigated combinations allow efficient discrimination of SP cells, indicating that DCV-based SP detection can be performed on various flow cytometric instruments without requiring change of filters.

## Abbreviations

CSC: Cancer stem cell
DCG: Vybrant DyeCycle Green
DCO: Vybrant DyeCycle Orange
DCV: Vybrant DyeCycle Violet
MFI: Median fluorescence intensity
NSP: Nonside population
SP: Side population
TSC: Tissue stem cell
UV: Ultraviolet.
