# Supplementary figures and images for: Optimized Stem Cell Detection Using the DyeCycle-Triggered Side Population Phenotype
Source: Stem Cells Int. 2015 Dec 20;2016:1652389. doi: 10.1155/2016/1652389 (PMC4699066; doi:10.1155/2016/1652389)

**A**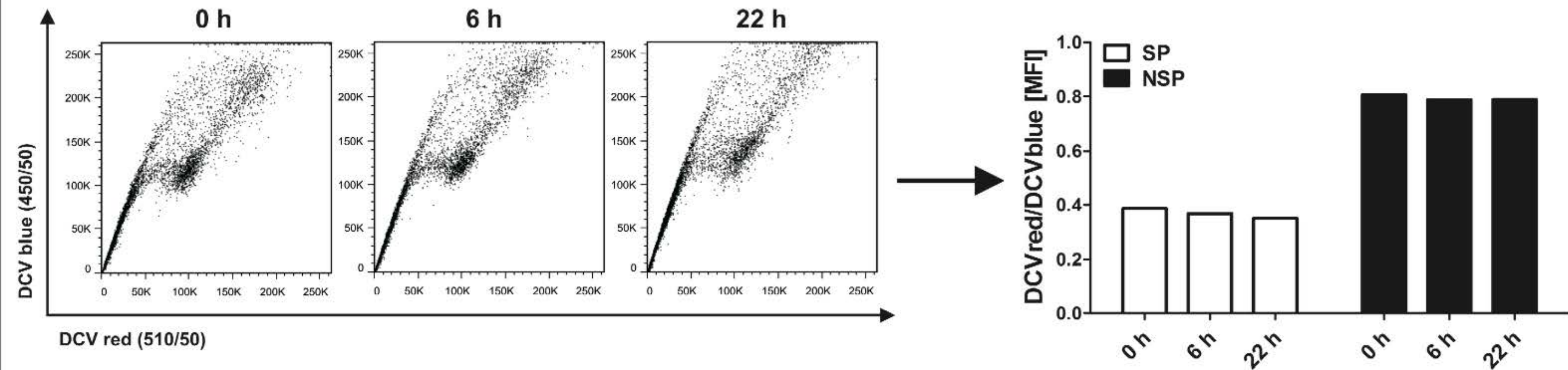**B**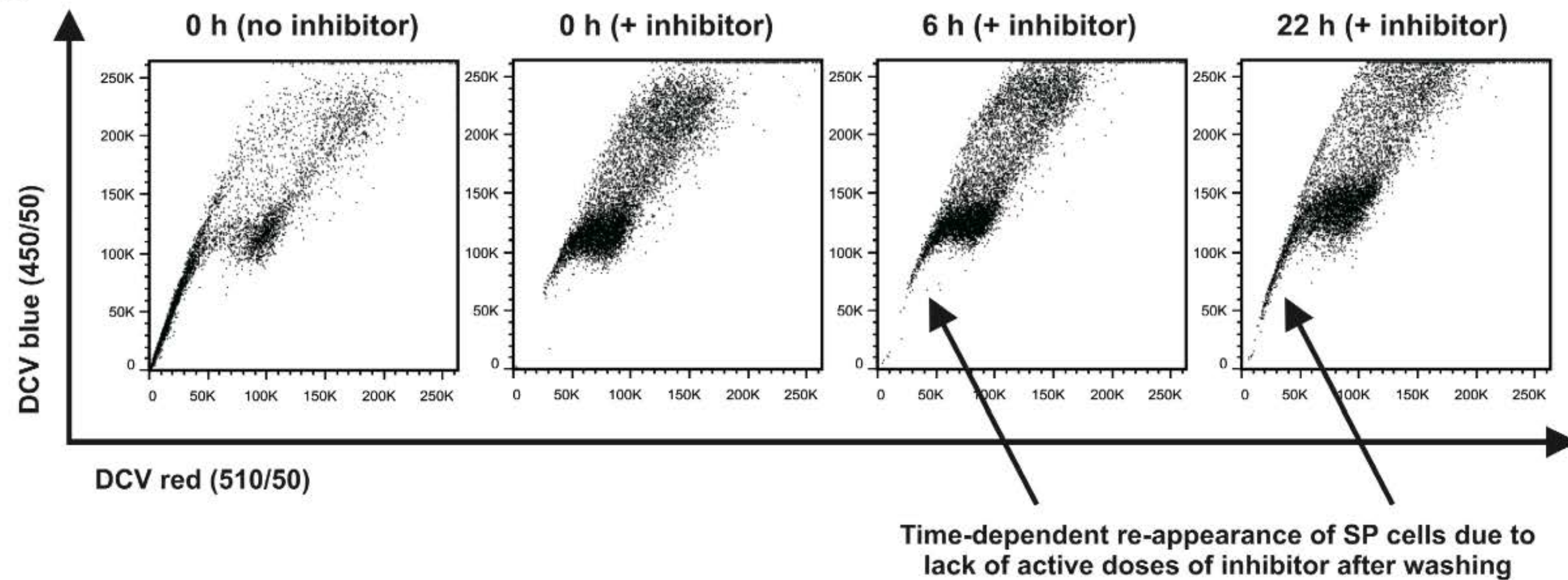

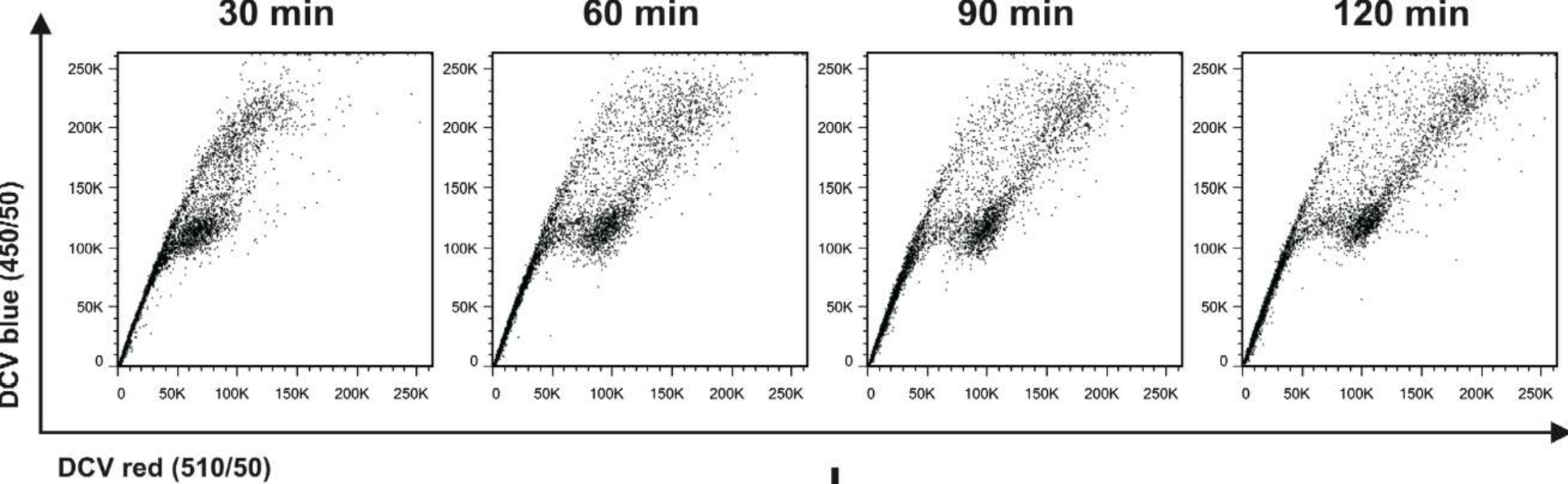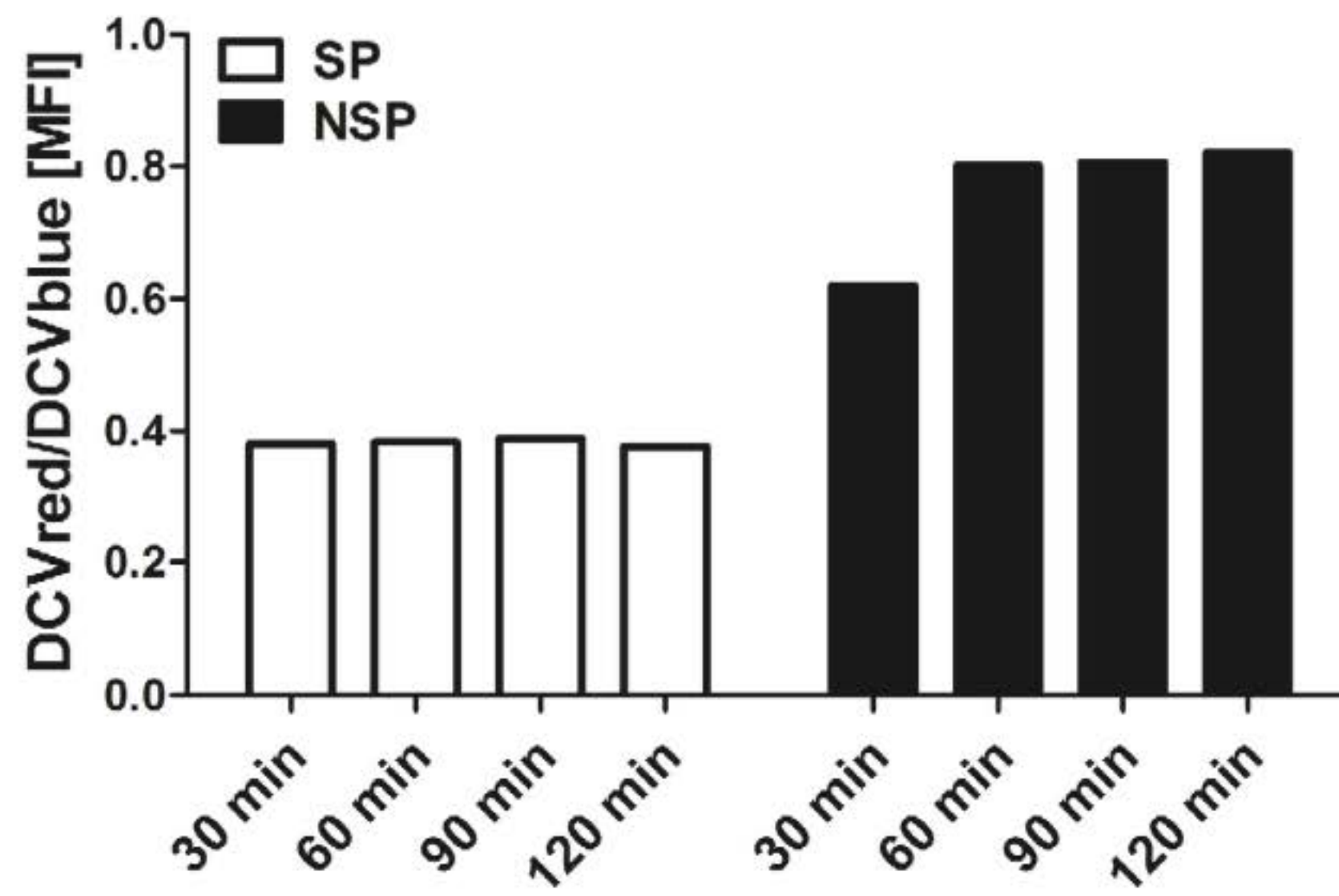

DCV blue (450/50)

no inhibitor

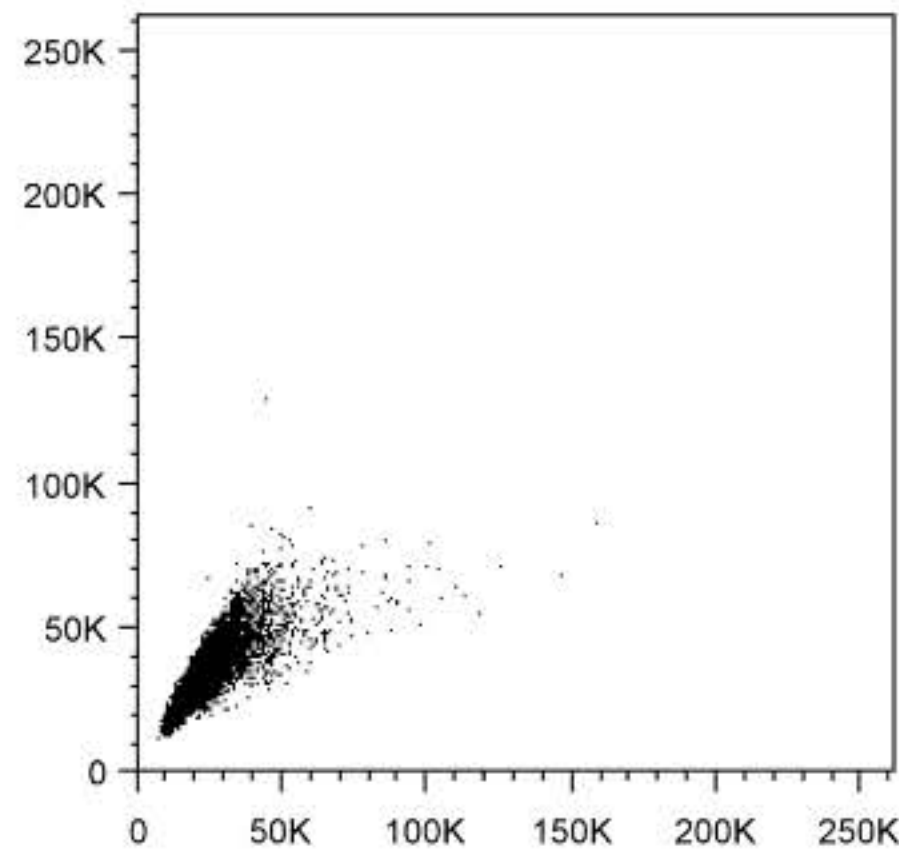

+ verapamil

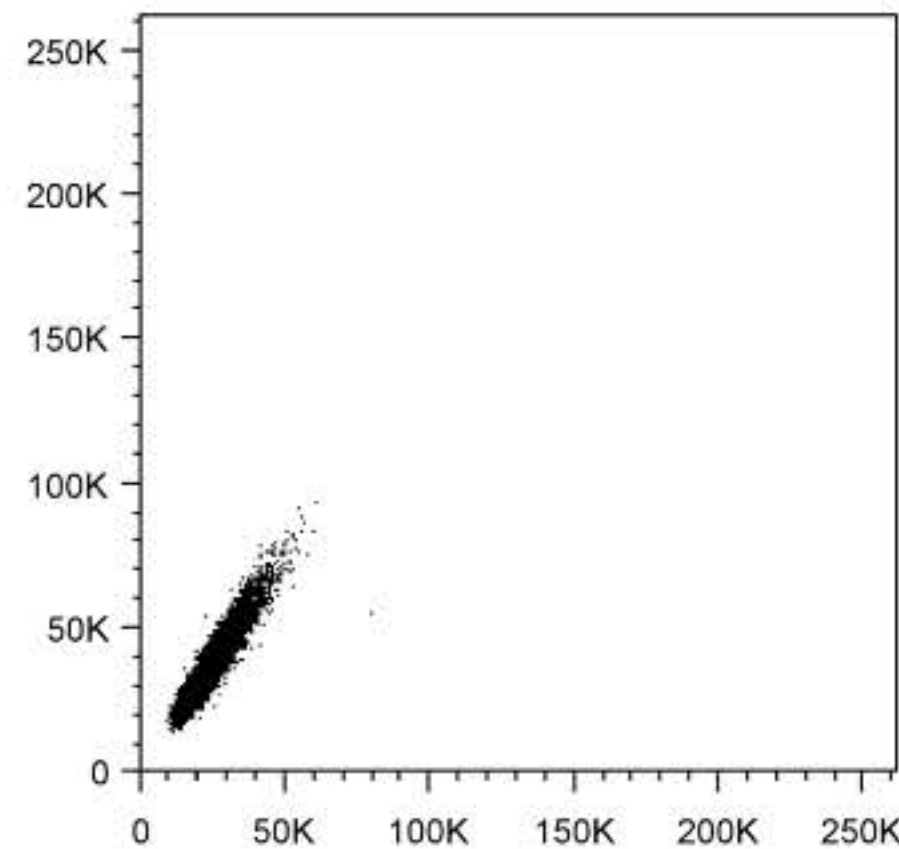

+ fumitremorgin C

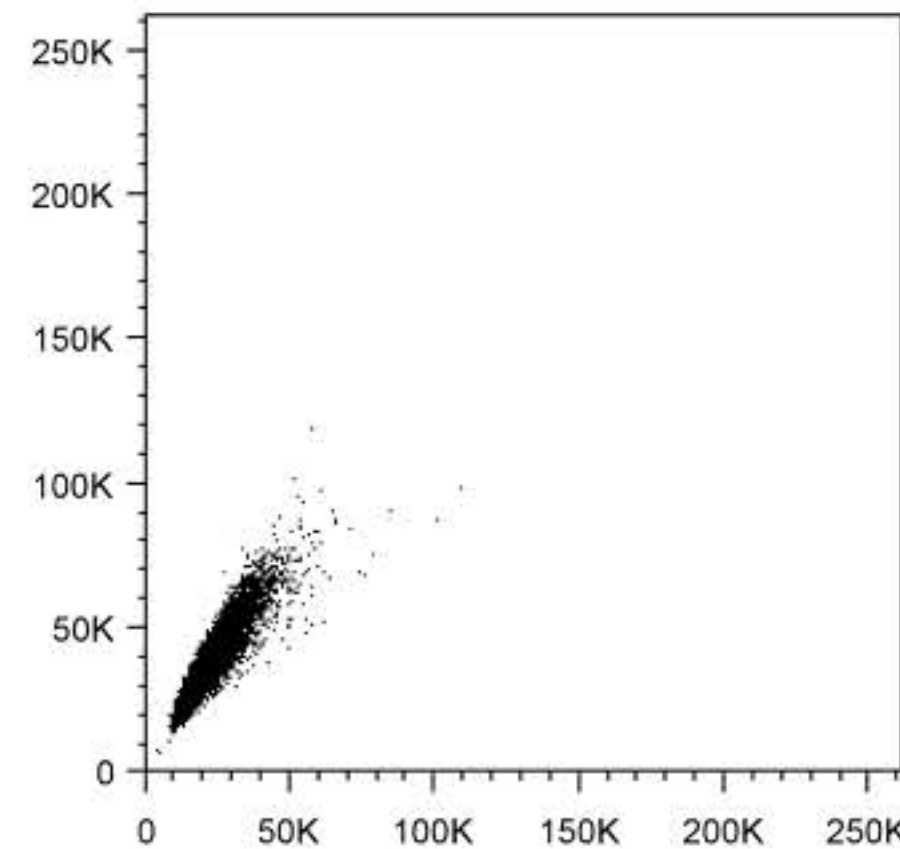

+ reserpine

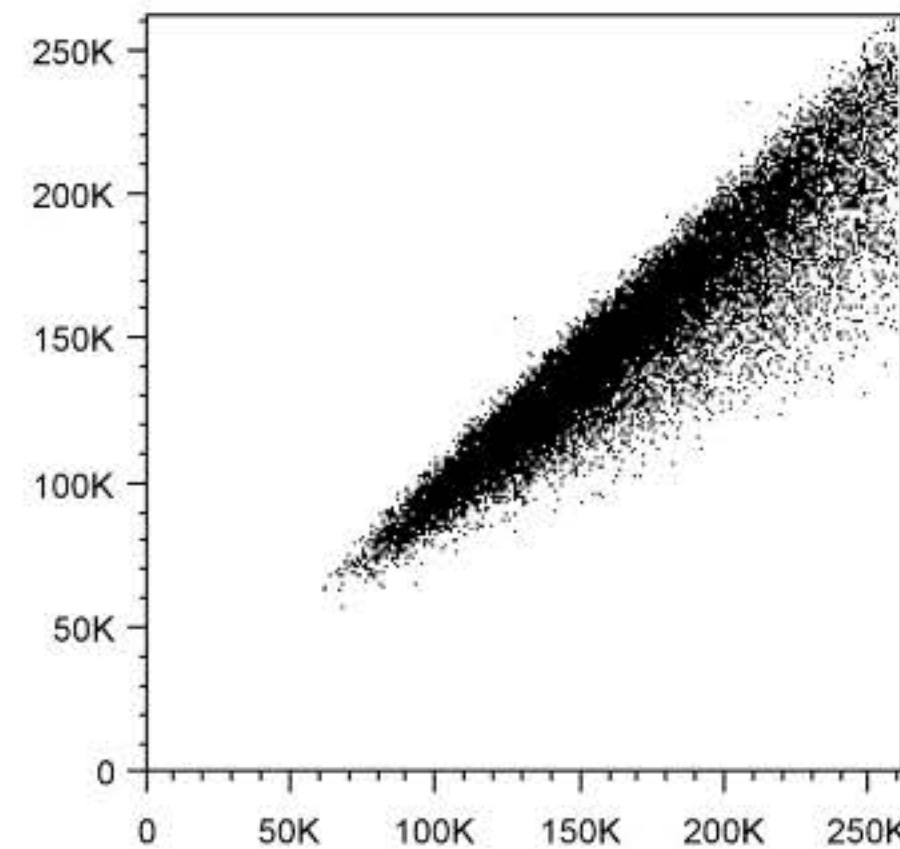

DCV red (510/50)

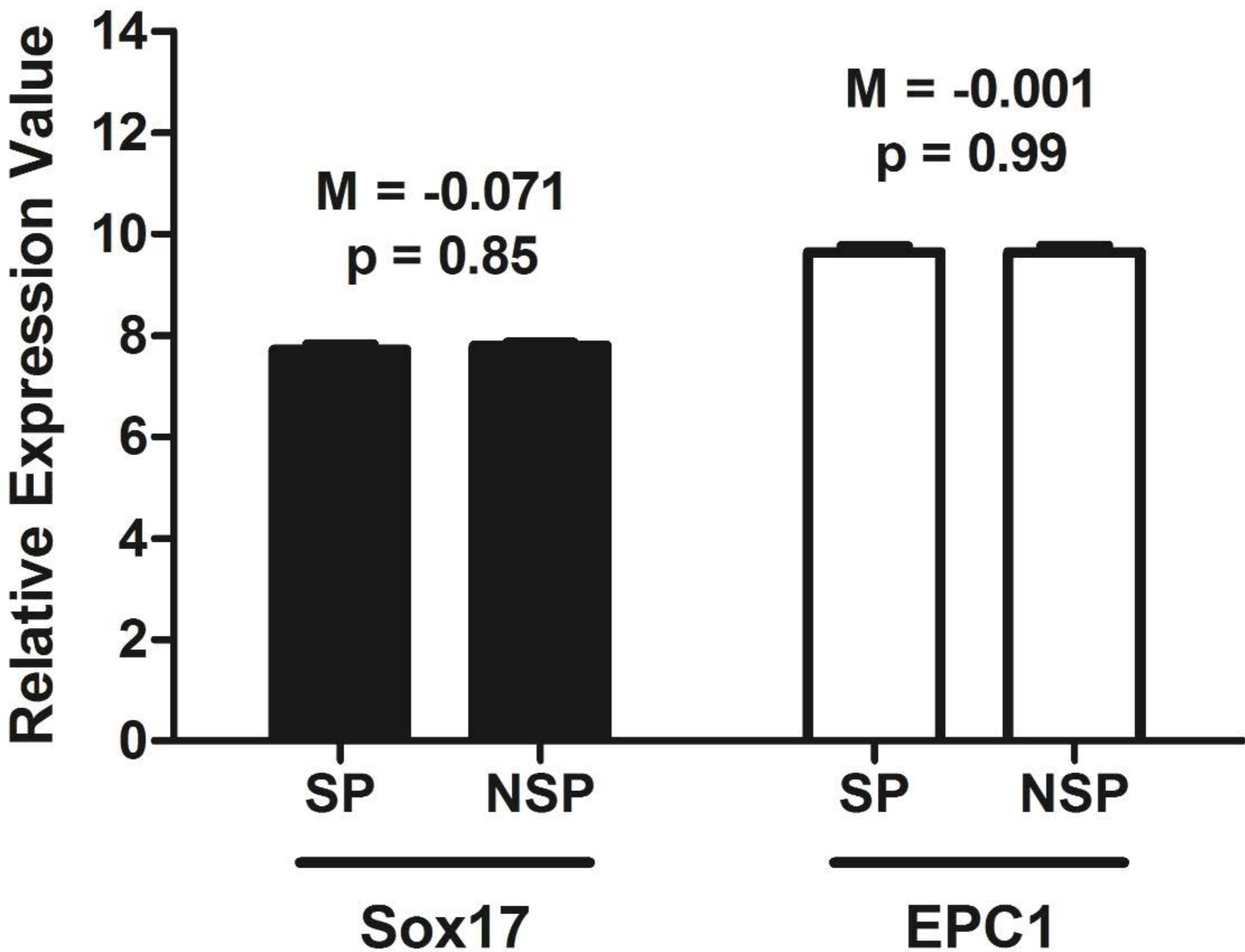

# LSRFortessa

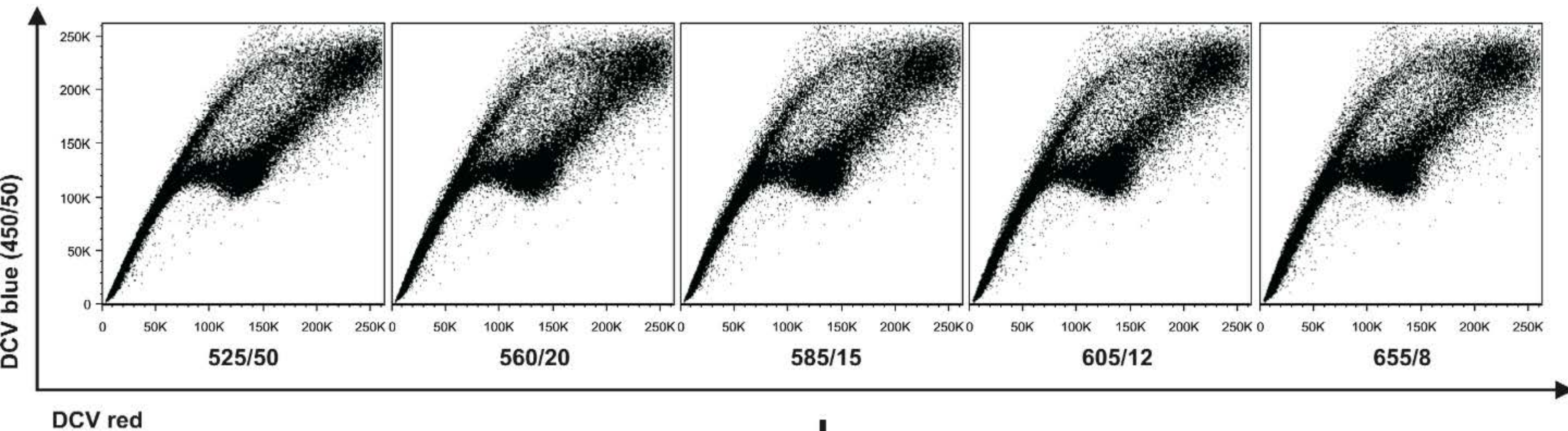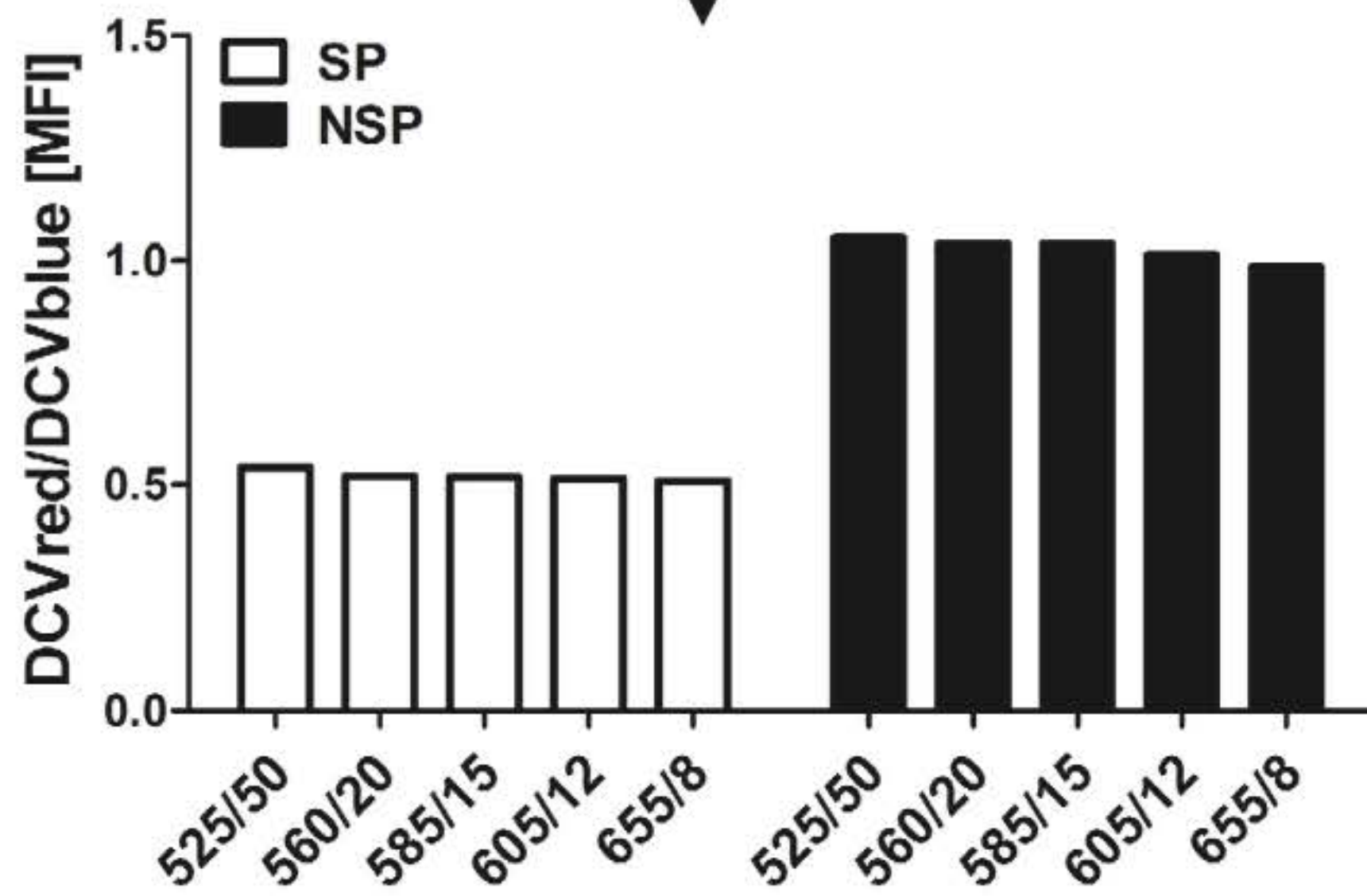

Supplement: Supplementary file 1 — Supplemental Figure 1: Stability of DCV-Stained Samples. (A) SP-enriched A2780 cells were stained with 10 µM DCV (106 cells/ml) and kept on ice in the dark for 0, 6 and 22 h prior to flow cytometry. Even though cell death is increased after 6 and 22 h (data not shown), the staining itself (i.e., the separation of SP from NSP cells) is stable over the investigated time period. (B) Kinetic analysis of the same cells inhibited with 20 µM fumitremorgin C. Note that after washing, SP cells gradually re-appear owing to the henceforth lack of active inhibitor. Supplemental Figure 2: SP/NSP Separation as a Function of Staining Duration. SP-enriched A2780 cells were stained with 10 µM DCV (106 cells/ml) for the indicated periods of time and analysed by flow cytometry. A staining duration of 30 min does not permit optimal dye accumulation in NSP cells, as detectable particularly in the long-wave range of DCV emission (‘DCV red'). Conversely, there is not much difference in staining outcomes between 60, 90 and 120 min, except that the NSP peak is somewhat sharpened with longer dye exposure. Altogether, and considering the heterogeneity in dye accumulation kinetics and cell death induction between different cell types, we propose a ‘default' staining duration of 90 min, which can of course be adapted upon demand. Supplemental Figure 3: Autofluorescence of Reserpine in the DCV-Relevant Wavelength Range. In the absence of DCV, A2780V cells were incubated for 90 min at 37°C with either no inhibitor, 50 µM verapamil, 20 µM fumitremorgin C, or 50 µM reserpine. Thereafter, cells were washed and analysed by flow cytometry for emission in the ‘DCV blue' (450/50) and ‘DCV red' (510/50) channels (note that due to the lack of DCV in the analysis, the detector voltages had to be increased accordingly). In contrast to verapamil and fumitremorgin C which are non-excitable by the violet laser, reserpine shows significant autofluorescence which might potentially interfere with the DCV signal, [file 1652389.f1.pdf]
